# Ventricular fibrillation immediately after the treatment of Graves’ disease coexisting with atypical angina and long QT syndrome: a case report

**DOI:** 10.1186/s13044-022-00136-2

**Published:** 2022-10-03

**Authors:** Hajime Iwasaki, Hirotsugu Suwanai, Hiroyuki Sakai, Keitaro Ishii, Natsuko Hara, Kazuhiro Satomi, Yasuyuki Takada, Yuki Nagamatsu, Ryo Suzuki

**Affiliations:** 1 Department of Diabetes, Metabolism, Endocrinology, Rheumatology and Collagen Diseases, Tokyo Medical University, 6-7-1 Nishishinjyuku, Shinjyuku-ku, Tokyo, 160-0023 Japan; 2 Department of Cardiology, Tokyo Medical University, Tokyo, 160-0023 Japan

**Keywords:** Graves’ disease, Beta-blockers, Typical angina, Long QT syndrome, ICD

## Abstract

**Background:**

Palpitations due to Graves’ disease are often caused by supraventricular arrhythmia. However, in rare cases, the background of coronary artery disease, genetic abnormalities, or channel abnormalities can cause ventricular fibrillation, which is a lethal arrhythmia. Here, we report a case of ventricular fibrillation after administration of beta-blockers early in the course of treatment for Graves’ disease coexisting with atypical angina and long QT syndrome.

**Case presentation:**

A 48-year-old man consulted a local general physician for chest discomfort and palpitations for approximately 2 weeks. He was diagnosed with Graves’ disease and treated with thiamazole 15 mg, bisoprolol 1.25 mg, and nitroglycerin 0.3 mg. The patient continued to experience chest discomfort the next day and visited our hospital. The patient was treated with landiolol 0.125 mg/kg/min for heart rate control, and 20 min later, electrocardiography showed a change from the R-on-T phenomenon to ventricular fibrillation. After cardiopulmonary resumption and improvement of thyroid function, a stress test was performed, which revealed coronary angina and long QT syndrome. An implantable cardioverter defibrillator (ICD) was implanted in the patient for secondary prevention. Since then, no fatal arrhythmia has been observed to date.

**Conclusions:**

When beta-blockers are administered to patients with Graves’ disease who have severe chest symptoms, fatal arrhythmias are possible. ICD implantation should be considered for the secondary prevention of fatal arrhythmias.

## Background

Graves’ disease is one of the most common forms of hyperthyroidism. The main symptoms include palpitations, sweating, and weight loss; most palpitations are supraventricular arrhythmias characterized by a narrow QRS during sinus rhythm [1]. However, in rare cases, a background of coronary artery disease, genetic abnormalities, or ion channel abnormalities may cause ventricular fibrillation (VF), which is a fatal arrhythmia. Here, we report a case of VF after early administration of beta-blockers for the treatment of Graves’ disease coexisting with atypical angina and long QT syndrome.

## Case presentation

The patient was a 48-year-old man with a history of surgery for seminoma at 38 years of age. The patient had no medical or family history of cardiac disease or sudden death. He reported a history of smoking 10 cigarettes per day between the ages of 18 and 20 years, with a smoking index of 200.

**Fig. 1:**
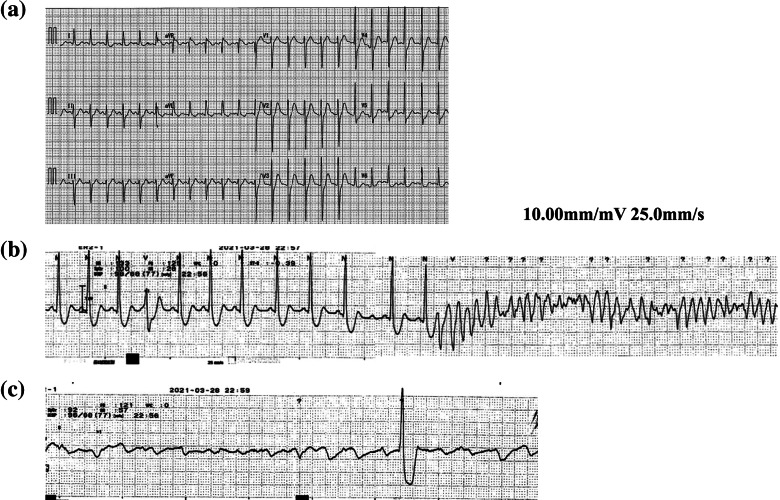
**a** Twelve-lead electrocardiogram on initial medical examination. **b** Electrocardiogram 10 min after administration of landiolol. Ventricular fibrillation was initiated by the R-on-T phenomenon of ventricular ectopic beat. **c** Electrocardiogram of ventricular fibrillation

The patient had experienced chest discomfort and palpitations for approximately 2 weeks prior to the emergency room (ER) visit at our hospital. He was diagnosed with Graves’ disease at another hospital the day before being admitted to our ER. Treatment with thiamazole 15 mg was initiated, along with the oral administration of bisoprolol 1.25 mg and nitroglycerin 0.3 mg for the chest symptoms and palpitations. On the day of ER admission, the patient experienced three events of chest discomfort. His chest symptoms improved at the first two times after treatment with nitroglycerin 0.3 mg. However, they did not improve during the third event, and he called for emergency medical care. On arrival at our ER, the patient’s Glasgow Coma Scale score was 15, and physical examination revealed a blood pressure of 122/72 mmHg and pulse of 142 beats/min. Electrocardiography (ECG) showed a narrow QRS and sinus tachycardia with ST depression in the precordial leads V4–V5 (Fig. 1). Laboratory findings were as follows: Mg, 1.8 (1.8–2.7) mg/dL; K, 4.6 (3.6–5.2) mEq/L; Ca, 9.2 (8.5–10.2) mg/dL; troponin T, 0.203 (< 0.1) ng/mL; brain natriuretic peptide, 93.2 (< 18.4) pg/mL; creatine kinase (CK), 180 IU/L; CK-MB, 18 IU/L; thyroid-stimulating hormone (TSH), < 0.01 (0.5–5.00) μIU/mL; free triiodothyronine (FT3), > 32.5 (2.3–4.3) pg/mL; free thyroxine (FT4), > 7.77 (0.9–1.7) ng/dL; TSH receptor antibody, 3.6 (< 2.0) IU/L; and thyroid-stimulating antibody, 122 (< 120) % (Table 1). We treated the patient with landiolol 0.125 mg/kg/min for heart rate control, and ECG showed a change from the R-on-T phenomenon to ventricular fibrillation (Fig. 1). He had a temporary cardiopulmonary arrest but resumed cardiopulmonary resuscitation (CPR) after several CPR sessions and one electric shock. The patient was admitted to the intensive care unit on the same day. Magnetic resonance imaging and contrast-enhanced computed tomography revealed no coronary artery stenosis or myocardial abnormalities. Thyroid ultrasonography showed diffuse goiter and increased blood flow in both lobes, which was consistent with Graves’ disease. The thiamazole dosage was adjusted according to the patient’s progress.

**Table 1:** Laboratory data

	Normal range	Adimission
Complete Blood Count		
White blood cell count (/µl)	2,700–8,800	6,500
Red blood cell count (/µl)	3.7–5.4 × 10^6^	4.76 × 10^6^
Platelet count (/µl)	140.0–340.0 × 10^3^	328 × 10^3^
Hemoglobin (g/dl)	11.0–17.0	11.9
Hematocrit (%)	34.0–49.0	41
Biochemistry		
Aspartate aminotransferase (IU/l)	8–38	23
Alanine aminotransferase (IU/l)	4–44	42
Lactate dehydrogenase (IU/l)	106–211	149
Creatine kinase (IU/l)	56–244	180
CK-MB (IU/l)	< 25	16
Sodium (mEq/l)	138–148	140
Potassium (mEq/l)	3.6–5.2	4.6
Chlorine (mEq/l)	98–108	101
Magnesia (mg/dl)	1.8–2.7	1.8
Calcium (mg/dl)	8.5–10.2	9.2
Blood urea nitrogen (mg/dl)	8.0–22.6	18.6
Creatinine (mg/dl)	0.4–0.8	0.69
Brain natriuretic peptide (pg/ml)	< 18.4	93.2
D-Dimer (µg/l)	< 0.80	0.98
Troponin T (ng/ml)	< 0.1	0.203
Thyroid-stimulating hormone (µlU/ml)	0.5–5.00	< 0.01
Free triiodothyronine (pg/ml)	2.3–4.3	> 32.5
Free thyroxine (ng/dl)	0.9–1.7	> 7.77
Thyroid stimulating hormone receptor antibody (IU/l)	< 2.0	3.6
Thyroid-stimulating antibody (%)	< 120	122

**Fig. 2:**
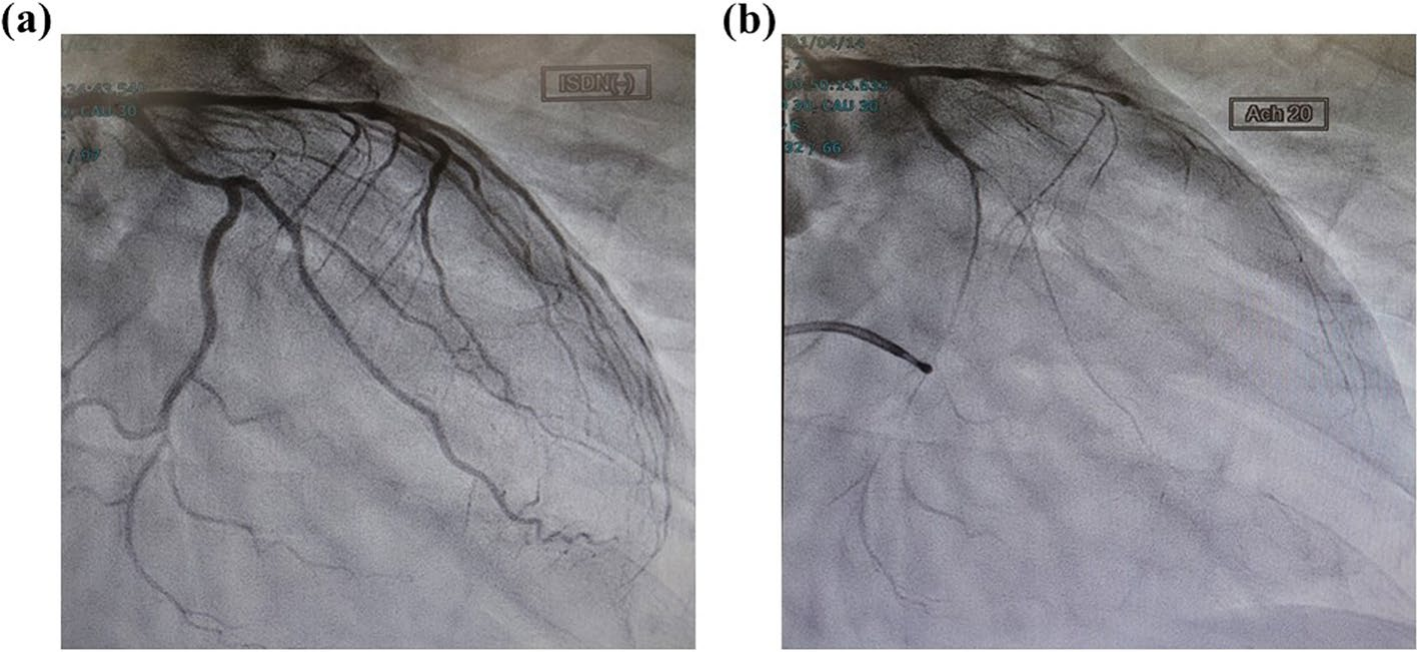
Acetylcholine (ACh) provocation test. **a** Left coronary angiography performed as a control before the Ach provocation test. **b** Injection of ACh 20 μg into the LCA provokes complete occlusion in the LAD

No signs of recurrent VF or other cardiac arrest rhythms were observed. When the patient’s general condition stabilized after improvement in thyroid function (FT3 5.09 pg/mL, FT4 1.91 ng/mL), exercise, epinephrine, and acetylcholine stress tests were performed. QT prolongation was not observed as a result of the exercise stress test with ergometer. In the epinephrine provocative test, QT/QTc was prolonged to 480/537 ms at 1 min after administration from 400/453 ms at baseline, suggesting a typical response of long QT syndrome type 2 (LQT2). ST changes and coronary artery stenosis were observed during the acetylcholine-loading test (Fig. 2). An implantable cardioverter defibrillator (ICD) was implanted on the 19th day for secondary prevention because there was a clear indication of atypical angina and QT prolongation syndrome.

## Discussion and conclusions

Beta-blockers are essential for heart rate control in thyrotoxicosis and are the first choice in the 2016 American thyroid association (ATA) guidelines [2]. However, a small number of case reportes fatal arrhythmias have been reported after administration of beta blockers to hyperthyroid patients [3, 4]. Our case suggests that beta-blockers cause VF in patients with thyrotoxicosis accompanied by coronary angina and subclinical long QT syndrome. Atrial fibrillation is a typical arrhythmia that complicates thyrotoxicosis in 5%–15% of patients [5]. In contrast, ventricular arrhythmias in patients with thyrotoxicosis are rare, and most VF cases are associated with hypokalemia. Kobayashi et al. proposed coronary angina as a mechanism of VF in thyrotoxicosis without hypokalemia [4]. In coronary angina, depolarization and repolarization abnormalities, as well as myocardial ischemia (angina pectoris attack) caused by coronary spasm, are observed, which increase the excitability of the ventricular muscle and eventually lead to fatal ventricular arrhythmia and sudden death [6]. In addition to stress and smoking, beta-blocker therapy has been reported to trigger coronary angina, where an overdose of beta-blockers may inhibit myocardial Na^+^ channels and cause wide QRS arrhythmias [7]. In our case, coronary angiography revealed an acetylcholine-induced vasospasm. Beta-blocker administration for heart rate control of thyrotoxicosis may have induced coronary angina that led to VF.

Long QT syndrome is another possible cause of VF. Most QT prolongation syndromes in Graves’ disease are caused by hypokalemia [8]; however, hypokalemia was not detected in our patient. Furthermore, FT4 and QT prolongation are positively correlated in patients with thyrotoxicosis [8]. In our case, significant QT prolongation was confirmed by an epinephrine stress test; congenital long QT syndrome was suspected, but the genetic test was negative.

In conclusion, our experience with this case suggests that the possibility of fatal arrhythmias should be considered when administering beta-blockers to patients with thyrotoxicosis accompanied by chest symptoms. Furthermore, ICD implantation should be considered for the secondary prevention of fatal arrhythmias.

## Data Availability

The datasets used and/or analyzed during the present study are available from the corresponding author upon reasonable request.

## Abbreviations

CPR: Cardiopulmonary resuscitation
ECG: Electrocardiography
ER: Emergency room
ICD: Implantable cardioverter defibrillator
VF: Ventricular fibrillation
LQT2: Long QT syndrome type 2
